# Exploring the Therapeutic Significance of microRNAs and lncRNAs in Kidney Diseases

**DOI:** 10.3390/genes15010123

**Published:** 2024-01-19

**Authors:** Luis Alberto Bravo-Vázquez, Sujay Paul, Miriam Guadalupe Colín-Jurado, Luis David Márquez-Gallardo, Luis Germán Castañón-Cortés, Antara Banerjee, Surajit Pathak, Asim K. Duttaroy

**Affiliations:** 1 School of Engineering and Sciences, Tecnologico de Monterrey, Campus Queretaro, Av. Epigmenio Gonzalez, No. 500 Fracc. San Pablo, Queretaro 76130, Mexicospaul@tec.mx (S.P.);; 2 Chettinad Academy of Research and Education (CARE), Chettinad Hospital and Research Institute (CHRI), Department of Medical Biotechnology, Faculty of Allied Health Sciences, Chennai 603103, India; 3 Department of Nutrition, Institute of Basic Medical Sciences, Faculty of Medicine, University of Oslo, N-0316 Oslo, Norway

**Keywords:** microRNAs, lncRNAs, kidney diseases, nephropathy, gene regulation, therapy

## Abstract

MicroRNAs (miRNAs) and long non-coding RNAs (lncRNAs) are two crucial classes of transcripts that belong to the major group of non-coding RNAs (ncRNAs). These RNA molecules have significant influence over diverse molecular processes due to their crucial role as regulators of gene expression. However, the dysregulated expression of these ncRNAs constitutes a fundamental factor in the etiology and progression of a wide variety of multifaceted human diseases, including kidney diseases. In this context, over the past years, compelling evidence has shown that miRNAs and lncRNAs could be prospective targets for the development of next-generation drugs against kidney diseases as they participate in a number of disease-associated processes, such as podocyte and nephron death, renal fibrosis, inflammation, transition from acute kidney injury to chronic kidney disease, renal vascular changes, sepsis, pyroptosis, and apoptosis. Hence, in this current review, we critically analyze the recent findings concerning the therapeutic inferences of miRNAs and lncRNAs in the pathophysiological context of kidney diseases. Additionally, with the aim of driving advances in the formulation of ncRNA-based drugs tailored for the management of kidney diseases, we discuss some of the key challenges and future prospects that should be addressed in forthcoming investigations.

## 1. Introduction

Kidney diseases, also known as renal diseases or nephropathies, refer to a group of medical conditions that affect the structure and function of the kidneys [1,2]. These diseases can affect one or both kidneys and can have various causes, including infections, genetic and epigenetic factors, autoimmune disorders, certain medications, high blood pressure, diabetes, and other underlying health conditions [3,4,5,6]. Some examples of common kidney diseases are chronic kidney disease (CKD), acute kidney injury (AKI), diabetic kidney disease (DKD), renal fibrosis, and kidney cancer [7,8]. The kidneys play vital roles, such as those in filtering waste products and excess fluids as well as toxins present in the bloodstream, maintaining an electrolyte balance, and regulating blood pressure [9,10]. Therefore, preventing and/or treating their damage and failure is paramount to avoid the onset of the abovementioned disorders.

The effectiveness of treatments for kidney diseases may vary according to diverse factors, e.g., the specific condition in question, the stage of the disease, and the patient’s overall health [11]. Early detection and intervention, along with proper management, could slow the progression of certain kidney disorders and alleviate symptoms. However, in some cases of advanced kidney disease or certain genetic conditions, treatments might be less effective, and patients may require more aggressive interventions like dialysis or kidney transplant [12,13]. Unfortunately, the scope of available treatments for kidney diseases remains limited. In addition, conventional pharmaceutical interventions for these diseases, such as steroids and immunosuppressants, are frequently associated with toxicity [14]. In such regard, numerous therapeutic approaches have arisen over the past years, including fecal microbiota transplantation [15], herbal medicines [16,17], telomere therapies [18], treatments targeting epigenetic modifications [19], immunotherapies [20], as well as sodium–glucose co-transporter 2 (SGLT2) inhibitors [21,22].

In particular, RNA-based therapeutic systems represent promising molecular tools for managing kidney diseases [23]. Among those tools, non-coding RNAs (ncRNAs) have been outlined as encouraging therapeutic targets for these life-threatening diseases [24,25,26]. NcRNAs represent a category of RNA molecules transcribed from DNA that do not encode proteins. Unlike mRNA, which acts as a template for protein translation, ncRNAs play diverse regulatory roles in the cell, controlling gene expression, chromatin structure, and various cellular pathways [27]. Consequently, ncRNAs are implicated in several biological functions, including development, cell differentiation, and disease mechanisms [28,29,30,31,32,33]. Two of the most essential types of ncRNAs are microRNAs (miRNAs) and long non-coding RNAs (lncRNAs), which can be found in specialized tissues as well as in the form of circulating ncRNAs in body fluids (e.g., plasma, serum, and urine) [34,35,36].

MiRNAs are a class of highly conserved, small, non-coding transcripts, typically consisting of about 20–24 nucleotides. They play crucial roles in post-transcriptional gene regulation by binding to specific mRNAs [37,38,39]. Predominantly, miRNAs exhibit imprecise complementarity to target mRNAs, and the degree of complementarity between the miRNA and the mRNA determines the mechanism through which the expression of the target gene is repressed (i.e., mRNA degradation or translation inhibition). Moreover, miRNAs are characterized by their high stability, which supports their use as biomarkers in the realm of clinical diagnostics [40,41,42]. Remarkably, miRNAs are involved in a wide range of biological functions, such as cell differentiation, cell growth, apoptosis, cell proliferation [43,44], tissue regeneration [45], immune responses, inflammation [46], and human reproduction [47,48].

Consequently, the aberrant expression of miRNAs has been associated with various human diseases, thereby making them an essential focus of research in the fields of molecular biology and medicine [49,50,51]. Due to their ability to regulate gene expression, miRNAs can influence critical molecular pathways involved in kidney function and pathology. Dysregulation of specific miRNAs is often observed in kidney diseases, contributing to disease progression. In such a context, some examples of miRNAs that are reported to be potentially involved in kidney diseases are miR-17, miR-21-5p, miR-22, miR-25, miR-29a/b/c, miR-30a-5p, miR-30d-5p, miR-30e-5p, miR-92a, miR-98-5p, miR-129-5p, miR-103a-3p, miR-145, miR-146a, miR-155, miR-192-5p, miR-192, miR-193, miR-194-5p, miR-196a-5p, miR-199a, miR-200, miR-205, miR-212-3p, miR-377, miR-584, miR-1285, and miR-1286 [52,53,54,55,56].

On the other hand, lncRNAs are a class of RNA molecules with a mean size longer than 200 nucleotides that do not encode proteins [57]. Indeed, lncRNAs exhibit distinctive features, including their extended length, the occurrence of a 3′ UTR, and an intricate intron/exon structure. Moreover, the expression of lncRNAs is typically lower than that of mRNAs, often manifesting in a species- and tissue-specific manner [58,59]. Moreover, lncRNAs display a notable scarcity of genomic variations, a characteristic akin to that observed in protein-coding genes [60]. Despite lacking a protein-coding capacity, lncRNAs play crucial regulatory roles in gene expression and various cellular processes. They interact with DNA, RNA, and proteins, modulating chromatin structure, transcription, and post-transcriptional regulation as well [61]. Owing to their broad ability to modulate gene expression, lncRNAs participate in multiple biological processes, including cell differentiation and development [62], programmed cell death [63], immune responses [64], bone metabolism [65], drug metabolism [66], and human reproduction [67]. As a result, the dysregulated expression of lncRNAs can trigger the development of multiple human ailments [68,69].

As a rapidly growing area of research, lncRNAs hold significant potential as key regulators and therapeutic targets in various biological and pathological contexts, including kidney diseases. For instance, taurine upregulated gene 1 (TUG1), metastasis-associated lung adenocarcinoma transcript 1 (MALAT1), *HOX* transcript antisense RNA (HOTAIR), HOXA cluster antisense RNA 2 (HOXA-AS2), myocardial infarction-associated transcript (MIAT), nuclear enriched abundant transcript 1 (NEAT1), HOXA transcript at the distal tip (HOTTIP), differentiation antagonizing non-protein coding RNA (DANCR), sepsis-induced kidney injury associated transcript 1 (SIKIAT1), small nuclear RNA host gene 5 (SNHG5), SNHG14, plasmacytoma variant translocation 1 (PVT1), ENSMUST00000147869, DKFZP43410714, Gm4419, NR_033515, 150Rik, 1700020I14Rik, long intergenic non-protein coding RNA 667 (LINC00667), LINC00963, LINC00968, and LINC01619 are lncRNAs that are associated with diverse kidney diseases [70,71,72,73]. It is worth noting that lncRNAs also work as endogenous sponges of miRNAs through a mechanism known as competitive endogenous RNA (ceRNA) regulation. Mechanistically, lncRNAs sequester miRNAs by forming interactions with them, thus diverting miRNAs from their target mRNAs and preventing miRNAs from exerting their inhibitory effect on mRNA expression. As a result, the abundance of target mRNAs increases, thus leading to altered gene expression and potentially influencing various cellular processes and disease pathways [74,75,76].

**Figure 1:**
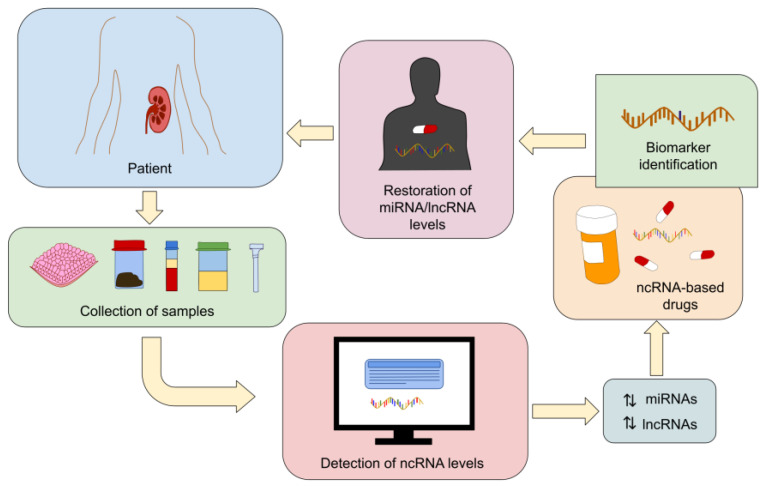
Prospective therapeutic uses for lncRNAs and miRNAs. Diagnosis procedures and ncRNA-based drugs for kidney diseases can be developed as a result of the identification of aberrantly expressed miRNAs and lncRNAs implicated in the clinical evolution of kidney diseases through the examination of various biological samples, including serum, renal tissue, urine, plasma, stool, and saliva.

Considering the key role of miRNAs and lncRNAs in the advancement of kidney diseases, next-generation ncRNA-based drugs aim to restore the expression levels of these transcripts. In cases where reduced miRNA expression underlies a given disease, the application of miRNA mimics (also known as agomiRs) offers a means to re-establish the levels of downregulated miRNAs. Conversely, anti-miRNAs (antagomirs) are employed to counteract the functions of disease-associated upregulated miRNAs by impeding their interaction with their mRNA targets [77,78]. Similarly, synthetic lncRNAs and anti-lncRNAs are harnessed to resettle the expression levels of downregulated and upregulated lncRNAs, respectively [79,80]. Additionally, harnessing lncRNA-miRNA interactions may open new possibilities for the development of innovative ncRNA-based therapeutics for a wide range of medical conditions, including kidney diseases and beyond (Figure 1).

Given the preceding insights, it is undeniable that both miRNAs and lncRNAs have remarkable potential as pharmacological targets for kidney diseases, offering potential alternatives to traditional treatments. Accordingly, this review provides a general perspective of recent significant studies regarding the therapeutic applications of miRNAs and lncRNAs in the molecular etiology and pathogenesis of kidney diseases. Moreover, since this research arena is still in its initial stages, here we discuss pertinent considerations that warrant attention in future research to enhance the management of kidney diseases through miRNA- and lncRNA-based approaches.

## 2. Therapeutic Inferences of miRNAs and lncRNAs in Acute Kidney Injury

Acute kidney injury (AKI) has become a major public health concern on a global scale since it is a life-threatening condition that lengthens hospital stays and causes the development of chronic kidney disease [81,82]. Recent research has revealed that several miRNAs display altered expression levels in urine and serum in AKI, suggesting that they could serve as valuable biomarkers and therapeutic tools for treatment of this disorder [83,84,85]. Additionally, AKI treatment might be aided by manipulating the expression levels of both lncRNAs and miRNAs [86]. Given the clinical significance of AKI in the worldwide population, the search for new-generation drugs has pointed to miRNAs and lncRNAs as potential targets for the management of this disease.

Zhang et al. [87] examined the mechanism of exosomes produced from endothelial progenitor cells (EPCs-exos) to ascertain how miR-21-5p/runt-related transcription factor 1 (RUNX1) axis contributes to sepsis-induced AKI. In the renal tissues of rats subjected to a cecal ligation and puncture (CLP) operation, a discernible decrease in the expression levels of miR-21-5p was evident coinciding with a concomitant upregulation in the levels of expression of the target of miR-21-5p, RUNX1. CPL rats that were injected with the miR-21-5p mimic showed elevated levels of miR-21-5p, which reduced serum inflammatory response, enhanced renal function, reduced pathological damage to the kidneys, decreased apoptosis as well as oxidative stress response in the kidneys, and controlled endothelial glycocalyx damage marker proteins syndecan-1 and heparanase-1. Additionally, rats that were injected with EPCs-exos (Exos and Exos^antagomir NC^) through a tail vein before a CLP operation displayed increased expression levels of miR-21-5p and had similar effects to those demonstrated when the levels of miR-21-5p were elevated in the previously mentioned CLP rats. Overall, this research indicated that EPCs secrete exosomes containing miR-21-5p to reduce sepsis-induced AKI by suppressing the expression of RUNX1 [87].

On the other hand, ischemia-reperfusion injury-associated RNA (IRAR) was found to be a putative lncRNA associated with AKI since it was primarily detected in tubular epithelial cells (TECs), which are tangled during the onset of AKI after ischemia-reperfusion (IR is the most typical cause of AKI) [88]. To determine the biological implications of IRAR in IR-induced AKI, both viral-based overexpression of IRAR and GapmeR-mediated silencing of IRAR were applied. In vivo, the silencing of IRAR in mice dramatically decreased the IR-induced infiltration of pro-inflammatory cells. In TECs, IRAR overexpression increased the expression of chemokines CXCL1/2 and CCL2 both at the transcript and polypeptide levels, but IRAR silencing decreased the levels of the same chemokines. The relationship between IRAR, CXCL1/2, and CCL2 was confirmed through RNA immunoprecipitation and an RNA pulldown assay. Moreover, inhibiting the CXCL1/2 chemokine receptor decreased neutrophil infiltration but had no obvious effects on kidney function. Overall, these outcomes reveal a novel role for IRAR in IR-induced AKI development by controlling chemokine production and immune cell infiltration, pointing to IRAR as a possible target for AKI prevention and/or attenuation [88].

Sepsis stands as the prevailing etiological factor underlying AKI. Analyses of plasma samples revealed that individuals afflicted by sepsis-associated AKI exhibited higher expression of the lncRNA small nucleolar RNA host gene 14 (SNHG14) [89]. Within LPS-stimulated HK-2 cells, SNHG14 demonstrated a diminishing effect on cell growth and autophagy, concomitant with an augmentation in cell death and the production of inflammatory cytokines. In this study, it was noticed that SNHG14 operated as a ceRNA, exerting negative modulation on the expression of miR-495-3p in HK-2 cells. Furter analyses revealed that miR-495-3p directly targeted the homeodomain interacting protein kinase 1 (HIPK1) in the aforesaid cell line. It was also observed that, following LPS stimulation, the interaction network involving SNHG14/miR-495-3p/HIPK1 orchestrates the regulation of HK-2 cell proliferation, autophagy, apoptosis, and the production of inflammatory cytokines. Additionally, by modulating NF-B/p65 signaling in LPS-challenged HK-2 cells, the SNHG14/miR-495-3p/HIPK1 network modulates the synthesis of pro-inflammatory cytokines, including TNF-α, IL-6, and IL-1. Consistently, SNHG14/miR-495-3p/HIPK1 may represent a potential therapeutic axis for the treatment of sepsis-induced AKI [89].

Small nuclear RNA host gene 5 (SNHG5) lncRNA expression is documented to be upregulated in the serum of individuals suffering from sepsis-induced AKI. Under such a premise, Wang and colleagues [90] reported that SNHG5 downregulation improves the viability of TCMK-1 and HK-2 cells challenged with LPS and decreases both the apoptosis rate and the generation of inflammatory cytokines. In addition, it was revealed that SNHG5 can interact with miR-374a-3p, which blocks nuclear factor-κB (NF-κB) activity by regulating the TLR4. Accordingly, the evidence presented in the study suggests that SNHG5 potentially modulates sepsis-induced AKI through the mediation of the miR-374a-3p/TLR4/NF-κB pathway, thereby offering novel insights into potential therapeutic strategies for this pathological condition [90]. 

In LPS-stimulated HEK293 and HK-2 cell models of AKI, the artificial overexpression of lncRNA cancer susceptibility candidate 2 (CASC2) using Lipofectamine 2000 improved cell survival and reduced cell death, epithelial-mesenchymal transition (EMT), migration, and oxidative stress [91]. Indeed, investigators discerned that CASC2 functions as a molecular sponge for miR-545-3p, thereby exerting control over the expression of peroxisome proliferator-activated receptor-α (PPARA), which is the mRNA target of miR-545-3p. On the other hand, the overexpression of miR-545-3p reversed the positive impact of CASC2 overexpression on an LPS-triggered injury in HEK293 and HK-2 cells. Furthermore, the overexpression of miR-545-3p exacerbated the cellular damage induced through LPS in both HEK293 and HK-2 cells, primarily by targeting PPARA. Overall, the overexpression of CASC2 reduced the harm caused through the LPS treatment applied to HEK293 and HK-2 cells by modulating the miR-545-3p/PPARA axis [91].

Another study elucidated that lncRNA 122049 prevented BUMPT cells (a cell line derived from the mouse proximal tubule) from entering apoptosis when exposed to IR. The observations of such an inquiry indicated that, mechanistically, lncRNA 122049 increases the expression levels of the ELK1 transcription factor by suppressing miR-330-5p expression [92]. In addition, through modulation of the miR-330-5p/ELK1 module, the upregulation of the lncRNA 122049 attenuated the advancement of ischemic AKI in mice through the modulation of the miR-330-5p/ELK1 axis. These results imply that lncRNA 122049 inhibits IR-induced kidney cell death by controlling the miR-330-5p/ELK1 module, showing novel insights into the etiology of ischemia AKI and suggesting a possible target for therapeutic intervention [92].

Recently, Xie et al. [93] reported that the expression of non-coding RNA activated by DNA damage (NORAD, a lncRNA) was upregulated in AKI mice models as well as in LPS-treated HK-2 cells. Suppressing the expression of NORAD reduced kidney damage by inhibiting apoptosis and inflammation in vivo. Additionally, NORAD deficiency prevented the apoptosis of HK-2 cells and reduced inflammation. In this investigation, scrutiny was directed toward elucidating the regulatory mechanism exerted by NORAD on HK-2 cells. Consequently, it was revealed that miR-577 directly interacts with NORAD, and GOLPH3 emerged as a downstream target of miR-577. Further assays demonstrated that the attenuation of apoptosis and inflammation resulting from NORAD silencing in vitro was reversed by the overexpression of GOLPH3. In conclusion, these authors showed that the suppressed expression of NORAD contributes to the reduction of renal injury in mice models of AKI and lowers apoptosis and inflammatory responses (triggered through exposure to LPS) in HK-2 cells by means of controlling the miR-577/GOLPH3 axis [93].

It is worth mentioning that AKI is a substantial risk factor for the onset of chronic kidney disease (CKD), and several miRNAs, such as miR-874-3p, were reported to control CKD significantly. In this regard, Yu et al. [94] induced epithelial cell injury using cisplatin in HK-2 cells and unilateral ureteral obstruction in C57BL/6 mice to scrutinize the functions and processes underlying the impact of human umbilical cord mesenchymal stem cell exosomes (HucMSC-Exos) on the restoration of renal tubular epithelial cells following injury. In particular, by targeting RIPK1, miR-874-3p, which was present within the HucMSC-Exo, controlled necroptosis and mitochondrial fission in renal tubular epithelial cells to support kidney damage repair. In summary, HucMSC-Exos might regulate necroptosis through miR-874-3p, thereby mitigating damage to renal tubular epithelial cells and fostering cell repair. Outstandingly, these outcomes introduce novel concepts for addressing AKI and impeding its progression to CKD [94].

**Figure 2:**
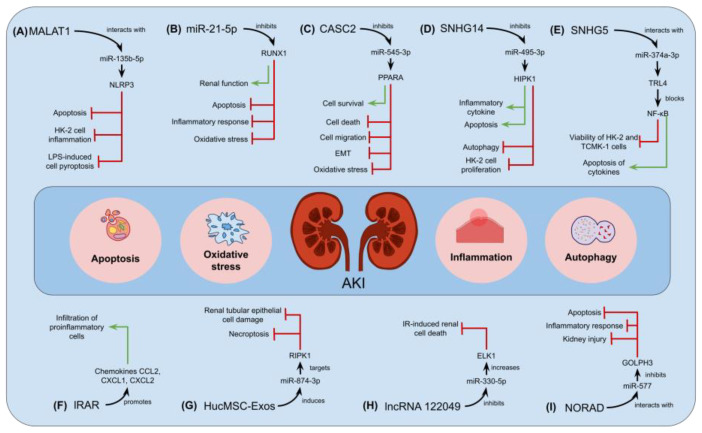
Schematic representation of the therapeutic implications of miRNAs and lncRNAs in AKI. In this comprehensive depiction, various miRNAs and lncRNAs play crucial roles in modulating different aspects of AKI. (**A**) The MALAT1/miR-135b-5p/NLRP3 signaling cascade influences LPS-induced cell pyroptosis, apoptosis, and inflammation. (**B**) MiR-21-5p, through exosome-mediated delivery, attenuates sepsis-induced AKI by suppressing RUNX1, leading to a reduction in serum inflammatory response, enhanced renal function, and diminished pathological damage. (**C**) CASC2, through the miR-545-3p/PPARA axis, mitigates LPS-induced damage in renal cells. The (**D**) SNHG14/miR-495-3p/HIPK1 axis and (**E**) SNHG5/miR-374a-3p/TLR4/NF-κB pathway contribute to the modulation of sepsis-induced AKI, influencing cell proliferation, apoptosis, autophagy, and inflammatory cytokine production. (**F**) IRAR, a lncRNA, emerges as a potential target for AKI prevention, controlling immune cell infiltration and proinflammatory responses. (**G**) MiR-874-3p, induced by HucMSC-Exos, controls necroptosis in renal tubular epithelial cells, offering a strategy to lessen cell damage and improve repair. (**H**) LncRNA 122049, by inhibiting IR-induced renal cell death through the miR-330-5p/ELK1 axis, demonstrates a potential therapeutic intervention for ischemic AKI. (**I**) NORAD, through the miR-577/GOLPH3 axis, reduces kidney injury, inflammatory response, and apoptosis in LPS-stimulated cells, highlighting the intricate regulatory network of miRNAs and lncRNAs in AKI therapeutics.

Huang and Xu [95] investigated how the lncRNA metastasis-associated lung adenocarcinoma transcript 1 (MALAT1) affects the human renal tubular epithelial HK-2 cells’ response to LPS-induced cell pyroptosis and inflammation. Mechanistically, the interaction between miR-135b-5p and MALAT1 was elucidated, revealing that MALAT1 positively regulated NLRP3 by acting as an RNA sponge for miR-135b-5p. Following gain- and loss-of-function assays, substantial evidence emerged indicating that both the depletion of MALAT1 and the upregulation of miR-135b-5p effectively inhibited LPS-triggered apoptosis, cell pyroptosis, as well as inflammation in HK-2 cells. In addition, the positive impact observed upon MALAT1 knockdown in HK-2 cells challenged with LPS was nullified through the suppression of miR-135b-5p expression. Remarkably, these observations represent novel insights into the activity of the MALAT1/miR-135b-5p/NLRP3 axis in the modulation of the inflammatory cell death induced through LPS [95] (Figure 2).

Noticeably, the current landscape of in vitro and in vivo studies investigating the roles of miRNAs and lncRNAs in AKI reveals notable limitations that warrant consideration. While these studies offer worthy insights into the molecular pathways behind AKI pathogenesis, a critical gap exists in the translation of these findings into clinical applications. The absence of robust clinical assays for the precise detection and quantification of specific miRNAs and lncRNAs poses a challenge in validating their diagnostic and prognostic potential. Additionally, concerns persist regarding the effective delivery of ncRNA-based drugs in a clinical setting. Issues related to stability, off-target effects, and achieving targeted therapeutic delivery to renal tissues remain substantial hurdles that need to be addressed to harness the full therapeutic potential of miRNA and lncRNA modulation in the context of AKI.

## 3. Therapeutic Inferences of miRNAs and lncRNAs in Chronic Kidney Disease

Chronic kidney disease (CKD) is a human illness distinguished by the gradual deterioration of kidney functions and afflicts approximately ten percent of the worldwide population. Most patients remain asymptomatic most of the time, presenting complications only in the advanced stages [96]. The principal causes of CKD are diabetes, chronic pyelonephritis, hypertension, and glomerular diseases. CKD has the potential to advance to renal failure and, in some cases, death [97,98]. Both miRNAs and lncRNAs hold considerable therapeutic promise for managing CKD. In fact, the unique regulatory capabilities of these ncRNAs position them as candidates for innovative therapeutic interventions and underscore their significance in the pursuit of effective treatments for CDK by targeting the key signaling pathways implicated in the progression of this disorder [99].

CKD significantly triggers cardiovascular problems, and one of the main particularities of people with CKD is left ventricular hypertrophy (LVH). An essential posttranscriptional regulator of LVH is miR-30. According to the findings of Bao et al. [100], CKD is associated with the poor expression of miR-30 in the myocardium. Thus, restoring the cardiomyocyte-specific miR-30 in CKD rats reduced LVH without affecting the development of CKD. It is significant to note that in cardiomyocytes miR-30 knockdown, which was transfected using an miR-30 sponge, employed both in vivo for 30SP mice and in vitro for H9C2 cells initiated cardiomyocyte hypertrophy by directly enhancing the calcineurin signaling. Furthermore, miR-30 transfection reduced the amount of cardiomyocyte hypertrophy brought on by CKD-related harmful substances, such as uremic toxin, angiotensin II, fibroblast growth factor-23, and transforming growth factor-β [100].

CKD causes a chronic and progressive condition called renal fibrosis. Exosomes made from MSC-Exos have been reported to decrease renal fibrosis and damage. Let-7i-5p was detected to be upregulated in both transforming growth factor β1 (TGF-β1)-stimulated NRK-52E cells and in mice kidneys following unilateral ureteral obstruction (UUO) [101]. Additionally, MSCs-Exos were used to deliver anti-let-7i-5p in order to lower the expression levels of the miRNA let-7i-5p within NRK-52E cells and raise the expression of tuberous sclerosis complex subunit 1 (TSC1), which is the target of the miRNA in question. Exosomal anti-let-7i-5p also hampered EMT in NRK-52E cells induced by TGF-β1 and in the kidneys of mice treated with UUO. When exposed to UUO, mice that had received exosomal anti-let-7i-5p showed reduced renal fibrosis and increased kidney function. Additionally, both in vivo and in vitro, exosomal anti-let-7i-5p prompted the activation of the tubular sclerosis complex subunit 1/mammalian target of rapamycin (TSC1/mTOR) signaling pathway, thus suggesting that exosomal let-7i-5p secreted by MSCs prevented renal fibrosis by activating the TSC1/mTOR pathway. In summary, these observations implicate that anti-let-7i-5p delivered through MSCs has anti-fibrotic activity in renal fibrosis models caused by UUO and TGF-β1 [101]. 

**Figure 3:**
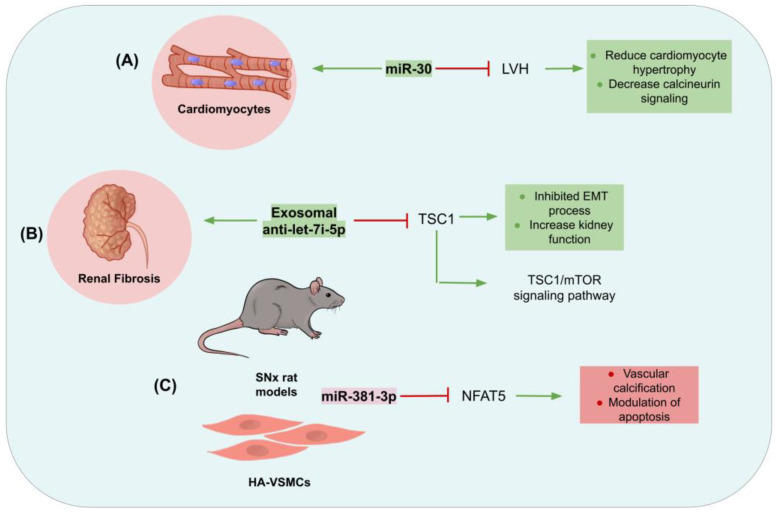
Mechanisms of action of ncRNAs in CKD. (**A**) miR-30 expression is downregulated in the myocardium in the presence of CKD, causing cardiomyocyte hypertrophy by increasing calcineurin signaling. (**B**) Exosomes reduce renal fibrosis by delivering anti-let-7i-5p, inhibiting the EMT process and prompting the activation of the TSC1/mTOR signaling pathway. (**C**) miR-381-3p was associated with apoptosis and calcification due to the decrease of miR-381-3p and NFAT5 (target gene of miR-381-3p) in high phosphate-stimulated human aortic vascular smooth muscle cells (HA-VSMCs) and 5/6 subtotal nephrectomy (SNx) rat models.

Cellular apoptosis belongs to the complex processes underlying vascular calcification (VC), a severe consequence of CKD. In research conducted by Liu et al. [102], high phosphate stimulated human aortic vascular smooth muscle cells (HA-VSMCs) and 5/6 subtotal nephrectomy (SNx) rat models were used to decipher the mechanism of exosomes generated from mesenchymal stem cells in the bone marrow (BMSC-Exos) [102]. Observations obtained in vivo and in vitro suggested that the impact of BMSC-Exo on the suppression of apoptosis and calcification was associated with the presence of miR-381-3p within the exosomes. Further analyses revealed that high levels of artery calcification in dialysis patients were correlated with diminished miR-381-3p expression and elevated nuclear factor of activated T cell 5 (NFAT5) expression, which is a target gene of miR-381-3p associated with apoptosis regulation. This investigation not only elucidates the substantial involvement of miR-381-3p/NFAT5 in the modulation of apoptosis and VC but also enhances comprehension of the regulatory impact of BMSC-Exo on conditions characterized by intricate mechanisms, such as CDK-associated VC, which might be helpful for developing novel ncRNA-centered treatments against this disorder [102] (Figure 3).

The current exploration of miRNAs and lncRNAs in CKD is crucial for understanding molecular complexities. However, substantial limitations exist, notably the absence of precise clinical assays for miRNA and lncRNA evaluation, hindering smooth translation of this understanding into clinical applications. In addition, concerns persist about the delivery of RNA-based drugs, encompassing stability, off-target effects, and targeted delivery challenges. Issues of drug toxicity and the reliability of biological models further underscore the need for careful consideration. Addressing these challenges necessitates a comprehensive approach, integrating advancements in clinical assays, delivery methods, and model systems to effectively bridge the gap between experimental insights and practical clinical applications for ncRNA-centered interventions in CKD.

## 4. Therapeutic Inferences of miRNAs and lncRNAs in Diabetic Kidney Disease

Diabetic kidney disease (DKD), also referred to as diabetic nephropathy (DN), is a type of chronic kidney disease and a common microvascular complication that is caused by diabetes mellitus. A recent study suggested that 40% of the population that is affected by diabetes mellitus, type 1 and 2, will develop diabetic kidney disease [103]. DKD stands as the predominant cause of end-stage renal disease (ESRD) globally. It is characterized by albuminuria (a marker of kidney disease that reflects a loss of the permselective barrier function of the glomerular capillary wall to macromolecules) and by progressive failure in the glomerular filtration rate (GFR) [104]. However, there are some limitations in the use of GFR for the proper diagnosis of DKD because it is not possible to directly measure the actual GFR in humans [104]. The progressive decline in renal function during DKD is caused by pathophysiological alterations in the kidneys, such as mesangial expansion, glomerular hypertrophy, and tubulointerstitial fibrosis, due to the accumulation of extracellular matrix (ECM) proteins, podocyte dysfunction, and basement membrane thickening [105]. Both miRNAs and lncRNAs exhibit substantial prognostic and therapeutic potential in DKD. Dysregulated expression of these ncRNAs plays a pivotal role in the intricate molecular pathways involved in DKD, influencing processes like inflammation and fibrosis. Consistently, these ncRNAs, with their gene-regulatory functions, might offer prospective alternatives for designing targeted approaches to modulate key pathways in DKD progression [106,107].

Early growth response 1 (Egr1) has been demonstrated to participate in fibrosis progression by interacting with the growth factor β (TGF-β)/Smad-dependent signaling pathway, which has a major role in the development of renal fibrosis and the inflammatory response in DKD. Moreover, miR-23a-3p is intricately involved in both inflammatory responses and the onset of diabetes. In order to elucidate the association between Egr1 and renal inflammation and fibrosis in DKD, Sheng et al. [108] evaluated the expression levels of Egr1, fibronectin (FN), and tumor necrosis factor-α (TNF-α) in renal tissues of DKD mouse models and HK-2 cells, discovering that they were upregulated both in vivo and in vitro, thereby suggesting that Egr1 can promote the expression levels of the fibrotic marker and inflammatory factors. Subsequently, the authors identified that miR-23a-3p targets Egr1 since their results showed that miR-23a-3p mimics reduced Egr1 expression. Finally, to demonstrate if miR-23a-3p regulates fibrosis and kidney inflammation through targeting Egr1, they co-transfected bovine serum albumin (BSA)-treated HK-2 cells with a miR-23a-3p inhibitor and a small interfering RNA targeting Egr1 (si-Egr1) revealing that miR-23a-3p inhibitor upregulated IL-6, TNF-α, and FN in HK-2 cells through Egr1. Additionally, the suppressed expression of Egr1 ceased the fibrosis and inflammation provoked through treatment using an inhibitor of miR-23a-3p. Thus, the inhibition of miR-23a-3p accelerated the expression of inflammatory fibrotic indicators and cytokines, demonstrating that miR-23a-3p may be crucial in the progression of DKD [108].

Kato et al. [109] noticed that the megacluster of miR-379 and the lncRNA lnc-megacluster (lncMGC) exhibit upregulation through endoplasmic reticulum (ER) stress in the kidneys of diabetic mice, instigating early features of DKD (e.g., hypertrophy and fibrosis). Subsequently, they designed a GapmeR (locked nucleic acid and phosphorothioate-modified antisense oligonucleotide) that inhibits the manifestation of both lncMGC and miR-379 clusters. Results demonstrated that inhibition of lncMGC through GapmeR reduced the expression of miR-379 and avoided the early manifestations of DKD in mice by averting ER stress. In addition, they exhibited that miR-379 targets the ER degradation enhancer mannosidase-like 3 (EDEM3). To analyze the regulatory activity of miR-379 in DKD and the putative DKD-related functions of the target genes of this miRNA (e.g., mitochondrial dysfunction, regulation of oxidative stress, and ER stress), miR-379KO mice were generated using the CRISPR-Cas9 nickase technique. In addition, mitochondrial fission 1 protein (FIS1), a crucial mitochondrial protein, was identified through AGO2-CLASH as one of the miR-379 targets. Upon further analysis, it was observed that adaptative mitophagy is regulated through FIS1 in mouse glomerular mesangial cells (MMC) and that the reduction of adaptive mitophagy through miR-379 overexpression results in diabetes, leads to reduced mitochondrial quality, and contributes to mitochondrial dysfunction [109].

Tubular injury is considered a significant factor in the early stages of DKD, and the intensity of kidney tubular damage is linked to renal activities. In this context, Cheng et al. [110] evidenced that miR-122-5p was upregulated in renal tubular cells in DKD, and it targets factor-inhibiting hypoxia-inducible factor-1 (FIH-1), which is of crucial importance since it blocks the activity of the hypoxia-inducible factor 1 subunit α (HIF-1α). To precisely understand the role of miR-122-5p in DKD, the effects of a miR-122-5p mimic were tested in streptozotocin (STZ)-induced diabetic nephropathy (DN) mice, and it was observed that the miR-122-5p mimic attenuated the tubular damage and interstitial fibrosis in DN mice, reduced the expression levels of urinary *N*-acetyl-β-d-glucosaminidase (NAG) as well as of the albumin-to-creatinine ratio (ACR), and diminished the expression levels of collagen I and vimentin. While anti-miR-122-5p increased urinary NAG and ACR levels, it aggravated tubular injury and renal fibrosis in STZ-induced DN mice. These observations suggest that miR-122-5p can potentially alleviate tubular injury in DN through the FIH-1/HIF-1α pathway [110].

**Figure 4:**
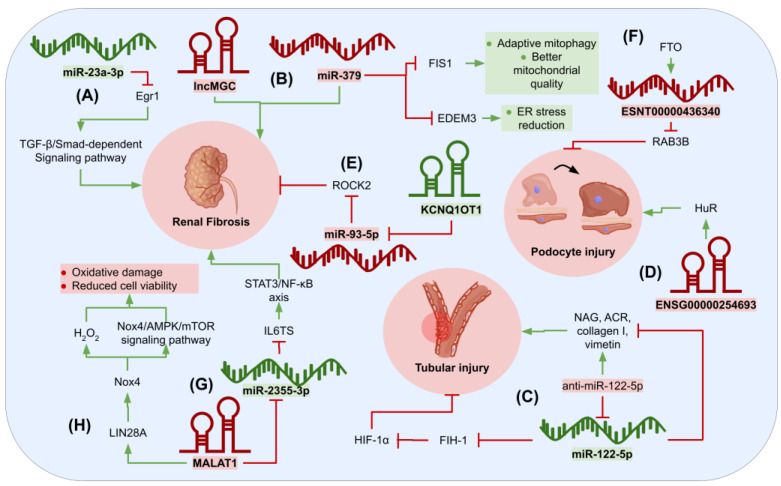
Graphical summary of the mechanisms of action of miRNAs and lncRNAs in DKD. NcRNAs with beneficial effects are colored in green, while those related to disease progression that could serve as therapeutic targets are colored in red. (**A**) MiR-23a-3p has been found to inhibit renal fibrosis by its effect on the TGF-β/Smad-dependent signaling pathway. (**B**) LncMGC and miR-378 have been associated with renal fibrosis, and the latter inhibits processes like adaptive mitophagy. (**C**) MiR-122-5p has been shown to lower levels of markers associated with tubular injury, possibly through inhibition of FIH-1. (**D**) ENSG00000254693, along with HuR, can lead to podocyte injury. (**E**) MiR-93-5p inhibits ROCK2, leading to fibrosis, but it is sponged by KCNQ1OT1. (**F**) ESNT00000436340 causes podocyte injury same through inhibition of RAB3B. (**G**) MALAT1 sponges miR-2355-3p, which inhibits the STST3/NF-κB axis, thus reducing fibrosis. (**H**) MALAT1 also leads to oxidative damage through activation of the Nox4/AMPK/mTOR signaling pathway.

Recent evidence indicates that the lncRNA ENSG00000254693 could bind to the human antigen R (HuR), a member of the embryonic lethal abnormal vision (ELAV) family of RNA-binding proteins that are involved in several diseases, such as colorectal and hepatocellular cancer, due to their crucial role in the regulation of mRNA splicing, stability, and transportation. Yu et al. [111] hypothesized that lncRNA ENSG00000254693 could interact with HuR to regulate podocyte injury in DKD, and the silencing of this lncRNA inhibits podocyte inflammation, apoptosis, and podocyte injury induced through high glucose (HG) conditions and the repression of HuR expression [111] (Figure 4).

Ongoing in vivo and in vitro investigations into the roles of miRNAs and lncRNAs in DKD are invaluable for deciphering underlying molecular complexities. However, a distinct shortage exists in the realm of clinical assays for precise quantification of these ncRNAs, thus impeding seamless translation of knowledge from these investigations into clinical applications. Moreover, concerns persist regarding the effective delivery of RNA-based drugs, encompassing issues of stability, potential toxicity, targeted delivery challenges, and reliability of biological models. These limitations emphasize the intricacies involved in moving from laboratory-based research to practical clinical applications and highlight the urgent need for interdisciplinary efforts to surmount these barriers and unlock the full therapeutic potential of miRNA and lncRNA interventions in the context of DKD.

## 5. Therapeutic Inferences of miRNAs and lncRNAs in Renal Fibrosis

Renal fibrosis occurs when wound healing mechanisms in the kidneys over function, leading to the accumulation of extracellular matrix (ECM) proteins in the kidney, which eventually cause organ injury and failure. This condition can be observed in patients with kidney impairments, particularly in advanced stages of CKD, and it is morphologically characterized by glomerulosclerosis, tubule atrophy, interstitial chronic inflammation, fibrogenesis, and vascular rarefaction [112]. Patients with renal fibrosis are required to change their lifestyles to slow down kidney damage. Such changes begin with alterations to their diet, weight, and physical activity, followed by specific therapy to delay disease progression. In ESRD, patients require renal replacement therapy or, if the former option is not available, palliative care [113]. Epigenetic therapies represent promising alternatives to slow down renal fibrosis progression as they can target pathways present in fibrotic niches, which refer to the microenvironments where scarring occurs [112]. Several ncRNAs, such as miR-29b [114], miR-122a [115], miR-21a-5p [116], lncRNA CRNDE as well as miR-29a-3p [117], and miR-26a-5p [118], have been shown to attenuate renal fibrosis. Therefore, they have the potential to be targeted through therapeutic approaches in order to treat renal fibrosis. 

Hu et al. [114] found that miR-29b suppressed the 3-kinase/protein kinase B (PI3K/AKT) pathway that is associated with EMT in renal fibrosis progression. They also noticed that the expression of miR-29b was downregulated in kidneys of CD-1 mice operated with UUO, which induced renal fibrosis, as well as in the rat proximal TEC line NRK-52E treated with angiotensin II (AngII) to induce a phenotype similar to that of renal fibrosis. Additionally, AngII-treated NRK-52E cells were transfected with a miR-29b mimic sequence, revealing that the expression of EMT-correlated α-smooth muscle actin (α-SMA), PI3K, and AKT decreased. In contrast, E-cadherin expression increased, which further proved EMT inhibition. On the other hand, the miR-29b inhibitor triggers the PI3K/AKT pathway and EMT. Although the authors did not perform in vivo studies of miR-29b delivery on UUO mice, their research could be expected to have effects that are similar to PI3K-inhibiting LY294002, which significantly reduced severe tubular dilatation, tubular atrophy, and widened interstitial space in mice kidneys. In summary, the results suggest that miR-29b has remarkable therapeutic potential for preventing renal fibrosis through PI3K/AKT inhibition [114].

Due to the growing evidence of the therapeutic potential of MSC-Exos against diverse diseases, interest in MSC-Exos miRNA’s role in renal fibrosis alleviation is currently increasing. In this context, Li et al. [115] analyzed the changes in the mTOR signaling pathway and autophagy responses to bone MSC-Exos as well as the impact of miR-122a mimic treatments on HK-2 cells (treated with TGF-β1 to induce a renal fibrosis-like phenotype) and male C57BL/6J UUO-operated mice. They noticed that miR-122a contained in bone MSC-Exos interferes with mTOR signaling mediating autophagy, which might be the cause of the antifibrotic activity presented by MSC-Exos and their therapy-relevant effects, such as a reduced expansion of the renal tubule, interstitial expansion, and preservation of kidney architecture [115].

In a subsequent study, Xu et al. [116] experimented with a different miRNA found in MSC-Exos: miR-21a-5p. When bone marrow MSC-Exos were administered to C57BL/6 UUO-operated mice, renal fibrosis was ameliorated, and knocking down miR-21a-5p from MSC resulted in a loss of their renoprotective activity. Further studies using UUO mice and TGF-β1-treated TCMK-1 cells indicated that miR-21a-5p mitigates renal fibrosis by repressing phosphofructokinase muscle isoform (PFKM) expression, therefore decreasing the rate of glycolysis in TECs and limiting the energy and substrates available for renal fibrosis progression. These experiments highlight the potential for future therapeutic strategies based on the antifibrotic and antiglycolytic properties of miR-21a-5p, which add up to the beneficial effects associated with MSC-Exos in the treatment of renal fibrosis [116].

Likewise, miR-26a-5p has been found to be downregulated in kidneys affected by fibrosis. Chung et al. [118] reported that intravenous miR-26a-5p restoration in UUO-operated mice suppressed the expression of markers associated with EMT, particularly TGFβ1 and the nuclear factor kappa-light-chain-enhancer of activated B cells (NF-κB), and reduced inflammation. In addition, in vitro experiments on NRK-49F cells and a human tissue microarray with renal fibrosis confirmed miR-26a-5p restoration in renal fibrosis tissue has antifibrotic and anti-inflammatory effects, showing its potential for developing ncRNA therapeutic approaches [118].

**Figure 5:**
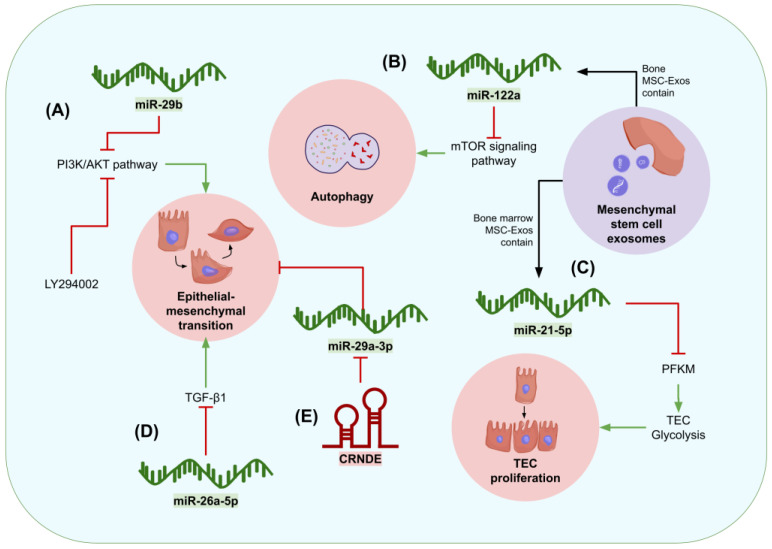
Graphical summary of the mechanisms of action of microRNAs and lncRNAs in renal fibrosis. NcRNAs with beneficial effects are colored in green, while those related to disease progression that could serve as therapeutic targets are colored in red. (**A**) MiR-29b has been found to inhibit renal fibrosis through its effect on the PI3A/AKT pathway, thus interfering with EMT, as observed through its similar effects to PI3K inhibitor LY294002. (**B**) MiR-122a, found in bone MSC-Exos, reduces autophagy and ameliorates renal fibrosis through inhibition of the mTOR signaling pathway. (**C**) Bone marrow MSC-Exos contain miR-21-5p, which reduces the glycolysis rate and slows down TEC proliferation. (**D**) MiR-26a-5p inhibits TGF-β1, which reduces EMT as well. (**E**) lncRNA CRNDE is associated with EMT progression as it targets miR-29a-3p.

The effect of lncRNA CRNDE (reported to be associated with fibroblast induction, inflammation, apoptosis promotion, and EMT in other diseases) on renal fibrosis was investigated by Zhao et al. [117]. In their study, UUO-operated Sprague Dawley rats and TGF-β1-treated renal epithelial cells were used to assess the expression of CRNDE, and it was observed that renal fibrosis-inducing surgery triggers the expression of this lncRNA. Moreover, CRNDE shRNA-mediated knockdown in UUO-operated mice resulted in a reduced expression of Collagen I, α-SMA, and vimentin related to EMT inhibition and renal fibrosis histological amelioration. Furthermore, it was determined that CRNDE targets miR-29a-3p since adding miR-29a-3p inhibitors to CRNDE knockdown cells reversed the effects on EMT signals discussed earlier (Figure 5). Accordingly, CRNDE lncRNA could be harnessed as a target for fibrosis therapies [117].

Although there might be missing elements in the current understanding of the interactions of miRNAs and lncRNAs with renal fibrosis, the conferred studies have provided a larger perspective of these complex phenomena. Clinical studies of miRNA- or lncRNA-based therapies are not yet available, and, while this technology is promising, there might still be challenges ahead [119]. Nonetheless, further research on the toxicity, doses, off-target effect and delivery of RNA-based drugs could be helpful in effectively managing renal fibrosis.

## 6. Therapeutic Inferences of miRNAs and lncRNAs in Autosomal Dominant Polycystic Kidney Disease

Autosomal dominant polycystic kidney disease (ADPKD) represents a monogenic inherited renal cystic disorder. It stands as the most prevalent genetic renal disorder, with an estimated prevalence ranging from 1 in 1000 to 1 in 2500 individuals. Its hallmark features include the formation of numerous renal cysts, resulting in substantial kidney enlargement and frequently progressing to renal failure during adulthood. The pathogenic basis of ADPKD lies in mutations occurring in either the *PKD1* or *PKD2* genes, which correspondingly encode the proteins polycystin-1 and polycystin-2. These proteins play a major role in cell physiology due to their influence on different biological pathways, including cell adhesion, differentiation, proliferation, and apoptosis [120,121]. It has been reported that patients suffering from ADPKD tend to have a reduced quality of life, increased psychological risk, reduced physical health, anxiety, and depression [122,123,124]. Currently, a vasopressin V2 receptor antagonist (V2RA) is the sole medication demonstrated to mitigate ADPKD disease progression. Nevertheless, the utilization of this drug is hindered by aquaresis-related adverse events, curtailing its broad application [125].

Despite the nascent stage of advancements in the field of ncRNAs concerning ADPKD, a few studies have illuminated the potential of both miRNAs and lncRNAs as viable therapeutic targets for addressing this genetic disorder. For instance, miR-30a-5p, miR-30d-5p, miR-30e-5p, miR-192-5p, and miR-194-5p exhibited notable downregulation in patients’ exosomes, murine *Pkd1* cystic kidneys, and human PKD1 cystic kidney tissue [126]. Similarly, miR-20a, miR-93-5p, and miR-106a-5p were detected as downregulated in serum samples from ADPKD patients who had undergone dialysis [127]. Altogether, these studies indicate that such miRNAs could be used as biomarkers and further studied for the development of miRNA-based drugs against ADPKD. Remarkably, Zhang et al. [128] elucidated that the DEAD-box RNA helicase p68 exerts repression on the *PKD1* gene expression through a miRNA-based post-transcriptional mechanism in renal epithelial cells. Mechanistically, p68 (along with the Drosha protein) serves as an upstream regulator facilitating the maturation process from pri-miR-17, pri-miR-200c, and pri-miR-182 to their respective precursor structures (i.e., pre-miR-17, pre-miR-200c, and pre-miR-182). In particular, researchers found that miR-182 represses the expression of *PKD1* through mRNA cleavage, contributing to the progression of ADPKD [128]. In summary, this report points out that both p68 and miR-182 may represent promising therapeutic targets for ADPKD.

**Figure 6:**
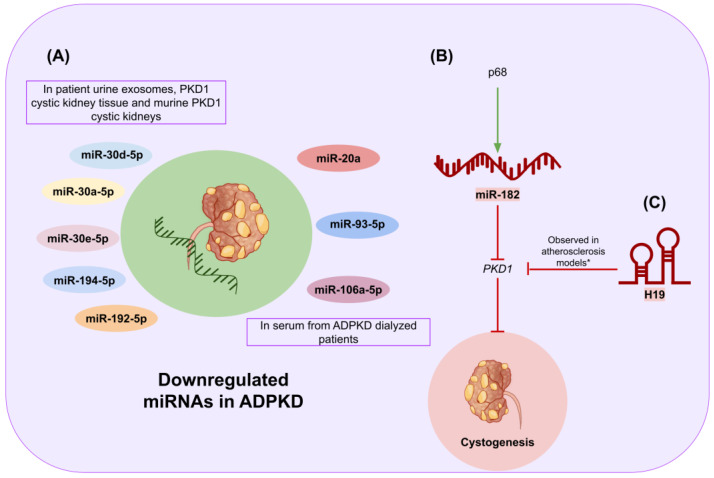
Graphical summary of the insights into the roles of microRNA and lncRNA in ADPKD. (**A**) Some of the miRNAs that have been found to be downregulated in samples associated with ADPKD are miR-30a-5p, miR-30d-5p, miR-30e-5p, miR-192-5p, miR-194-5p, miR-20a, miR-93-5p, and miR-106a-5p. Some mechanisms of action of ncRNA are also presented. NcRNAs with beneficial effects are colored in green, while those related to disease progression that could serve as therapeutic targets are colored in red. (**B**) p68 facilitates the maturation process of miR-182, which has been found to repress *PKD1*, which in turn can lead to cystogenesis. (**C**) LncRNA H19 silencing has been associated with *PKD1* upregulation (* this regulatory role has not yet been studied in models of ADPKD; however, findings obtained in atherosclerosis models might provide novel insights to target H19 in ADPKD).

On the other hand, Yang et al. [129] reported that silencing the lncRNA H19 upregulates the expression of *PKD1* in the context of atherosclerosis. Although this investigation was concentrated on a disease different from ADPKD, the methodologies employed may all the same be extrapolated to develop and assess a possible treatment for ADPKD through the silencing of H19 (Figure 6). Nonetheless, additional studies are imperative to discern specific miRNAs and/or lncRNAs that could serve as optimal therapeutic targets for this particular disease.

## 7. Therapeutic Inferences of miRNAs and lncRNAs in Renal Cancer

In 2018, kidney cancer was estimated as the cause of 2.2% of new global cancer cases, out of which renal cell carcinoma (RCC) is the most frequent one [130]. RCC encompasses a variety of cancers that affect renal tubular epithelial cells of which clear cell RCC (ccRCC) is the most common variation. Early symptoms may result from immune responses or hormone and cytokine excretion by the tumor, including hypercalcemia, fever, and erythrocytosis, while patients may experience flank pain, gross haematuria, and a palpable abdominal mass in advanced untreated cases. Regardless of the tumor’s stage, up to 77% of patients experience anxiety, and reports on metastatic renal cell carcinoma patients showed that pain, fatigue, and financial comorbidity are the most common distress contributors [131,132,133].

This type of cancer is usually resistant to common chemotherapy and radiotherapy, which highlights the importance of devising novel therapeutic approaches. It has been previously noted that both lncRNAs and miRNAs are involved in the regulation of carcinogenesis, tumor progression, and metastasis. This is also true for RCC, where miRNA/lncRNA axes have been identified as potential biomarkers and regulators [134,135]. One such lncRNA involved in RCC is LINC00641, which has been previously associated with tumorigenicity in different types of cancer. Zhang et al. [136] performed qRT-PCR on 48 RCC tissue samples collected from adult patients who had undergone nephrectomy, discovering that LINC00641 is upregulated in RCC and might be related to patient survival. These results were further validated by comparing HK-2 normal kidney cells with RCC cell lines. Following tests were performed on the RCC cell lines A498 and ACHN as well as on BALB/c nude mice, where RCC was induced by grafting using ACHN cells, which in turn showed that the shRNA-mediated depletion of LINC00641 suppressed RCC cell growth in vitro and tumorigenesis in vivo. Additionally, through bioinformatic analysis and the luciferase reporter assay, it was determined that LINC00641 is a ceRNA for miR-340-5p and that inhibition of this miRNA restored RCC cell proliferation and invasion in vitro, suggesting that the LINC0064/miR-340-5p axis is relevant to RCC tumorigenesis [136].

Later, Guo et al. [137] inferred the importance of renal cancer-associated transcript 1 (RCAT1) lncRNA in RCC through bioinformatic methods. They observed an upregulation in RCAT1 both in the RCC tissue of 52 ccRCC nephrectomy patients and in RCC cell lines, remarking that it facilitated the proliferation, migration, and invasion of RCC cell lines 786-O and 769-P. Interaction between RCAT1 and miR-214-5p was confirmed using the luciferase assay, which in turn was found to interfere with cell cycle regulation-associated transcription factor E2F2 through binding to the 3′UTR region of its mRNA. Following these results, BALB/c nude mice were used in a xenograft model, which showed that RCAT1 knockdown reduced RCC proliferation, as evidenced through significatively smaller tumor weight and volume gain over 30 days and through the reduction of cell proliferation marker antigen Kiel 67 (Ki67) and E2F2 according to qRT-PCR and immunohistochemistry. Finally, a lung metastasis mouse model was used to show that RCAT1 knockdown diminished the size and number of metastatic lesions in the mice lungs after 4 weeks of grafting [137]. These results indicate that targeting RCAT1 might help reduce tumor proliferation and metastasis for RCC, and miR-214-5p mimic administration could also be explored in the future.

Hypoxic microenvironments have been associated with tumor progression and metastasis. By testing knockdown and overexpression of LOC100506178 in Caki-1 and ACHN cells, it was found that this lncRNA could positively modulate RCC invasion and migration as well as induce EMT in both low and normal oxygen conditions alike. It was also noticed that LOC100506178 acts as a ceRNA by using fluorescence in situ hybridization (FISH) to determine co-localization with miR-613, miR-206, and miR-1-1-3p, which was confirmed through a luciferase assay [138]. After this, it was suggested that miR-613, miR-206, and miR-1-1-3p target Jagged1 (JAG1) in ACHN and Caki-1 cells transfected with mimics for all three of these miRNA. JAG1 is a ligand for the Notch signaling pathway that regulates the C-X-C chemokine receptor type 4 (CXCR-4) that mediates cell invasion and metastasis. BALB/c nude mice were used in an orthotopic xenograft model with SN12PM6 cells. LOC100506178 overexpression resulted in a higher distant metastasis rate and shorter overall survival, while CXCR-4 CRISPR knockdown diminished lung metastasis rate and reversed pro-metastatic effects when combined with LOC10050178 overexpression [138]. These observations suggest that downregulating CXCR-4 through the Notch signaling pathway can be the basis for an antimetastatic therapy for RCC and LOC10050178 could be a relevant target to attain this goal.

A protein-coding-esterase domain containing 1B antisense RNA 1 (PCED1B-AS1) lncRNA was found to be upregulated in the ccRCC tissue of 40 nephrectomy patients, and its increased expression was correlated with a higher tumor state, as reported by Qin et al. [139]. To further elucidate the role of this lncRNA in RCC, PCED1B-AS1 expression was knocked down in A498 and Caki-1 cells, leading to the inhibition of kidney cancer cell proliferation. In addition, it was determined that PCED1B-AS1 is expressed in the cytoplasm, suggesting that it is a ceRNA associated with miR-484. This lncRNA-miRNA module was then confirmed through luciferase and immunoprecipitation assays. In addition, miR-484 was demonstrated to possess tumor-suppressing activity in A498 and Caki-1 cells transfected with a miR-484 mimic, which presented reduced RCC cell proliferation and migration. It was determined that it did so by inhibiting zinc finger E-box-binding homeobox 1 (ZEB1) expression, which is a regulatory element of the transcription factor network and is substantially responsible for EMT control [139]. This information suggests that PCED1B-AS1 inhibition and miR-484 mimic administration could be used as strategies to deter RCC progression.

As lncRNA cytoskeleton regulator (CYTOR) has been functionally associated with a variety of tumors, Wang et al. [140] used ACHN, Caki-1, Caki-2, and 786-O cell lines to assess its expression in RCC models, finding that it is upregulated while miR-136-5p is downregulated. Afterward, through a luciferase assay, CYTOR was confirmed to sponge miR-136-5p. Inhibiting the expression of CYTOR minimized cell proliferation and promoted apoptosis in 786-O and Caki-1 cells. MiR-136-5p was found to regulate the expression of the methionine adenosyltransferase 2B (MAT2B) oncogene, whose protein product interacts with Bcl-2-associated anti-apoptotic protein 3 (BAG3), promoting the proliferation, migration, and invasion of RCC cells. Finally, in vivo studies showed that CYTOR knockdown led to an increase in miR-136-5p expression and RCC inhibition [140]. 

As evidenced in the previous lines, ncRNAs have great importance in the development and progression of RCC. Each of the ones discussed in this section has a distinctive role in the disease, showcasing a remarkable variety of gene and pathway targets that also speak to the complexity of RCC; however, the single-molecule focus of most publications might fail to paint a full picture of the ncRNAs as RCC treatments, potentially ignoring the effects of non-specific delivery or off-target action. Most of the current research on RCC-relevant ncRNAs is carried out on ccRCC models that do share characteristics with other RCC types. These reports show promising ncRNA targets and mechanisms to diagnose and treat RCC, but it is worth noting that their foremost purpose was in revealing and evidencing these molecular interactions and not in designing and testing a therapy suitable for human trials. Accordingly, there is still a great window of opportunity regarding the clinical translatability of ncRNA-based drugs against the different types of RCC.

## 8. Concluding Remarks

In recent years, significant studies have shed light on the critical regulatory functions played by miRNAs and lncRNAs in the pathogenesis of kidney diseases. It has been demonstrated that the dysregulated expression of these ncRNAs impacts various pathological processes associated with the onset and progression of kidney diseases, encompassing renal tubular epithelial cell injury, renal vascular changes, podocyte and nephron loss, renal fibrosis, sepsis, inflammation, pyroptosis, apoptosis, among others. The experimental evidence presented in this review underscores the remarkable clinical prospects of miRNAs and lncRNAs as therapeutic agents against kidney diseases. However, it is crucial to acknowledge that comprehensive investigations are necessary to fully comprehend the enigmatic modulatory roles of miRNA and lncRNA transcriptomes that continue to elude our understanding of kidney diseases’ etiology. Moreover, additional pre-clinical and clinical studies are necessary to guarantee the efficacy and safety of the miRNA- and lncRNA-centered therapeutic approaches discussed herein.

## 9. Future Insights

As shown in the preceding sections, biologists have diligently scrutinized the therapeutic relevance of miRNAs and lncRNAs in the context of kidney diseases. Notwithstanding this, several lingering concerns necessitate thorough exploration in forthcoming research endeavors. It is paramount to rigorously assess the potential adverse effects, pharmacodynamics, pharmacokinetics, and toxicity profiles of miRNA- and lncRNA-based drugs for kidney diseases [141,142,143]. In this regard, a comprehensive understanding of these factors is indispensable in order to validate the safety and efficacy of such novel therapeutic approaches. By meticulously investigating how these ncRNA-based drugs interact with the cellular machinery, impact gene expression, and influence various physiological processes, scientists can mitigate unforeseen risks and pave the way for developing precise and well-tolerated therapies against kidney diseases.

**Figure 7:**
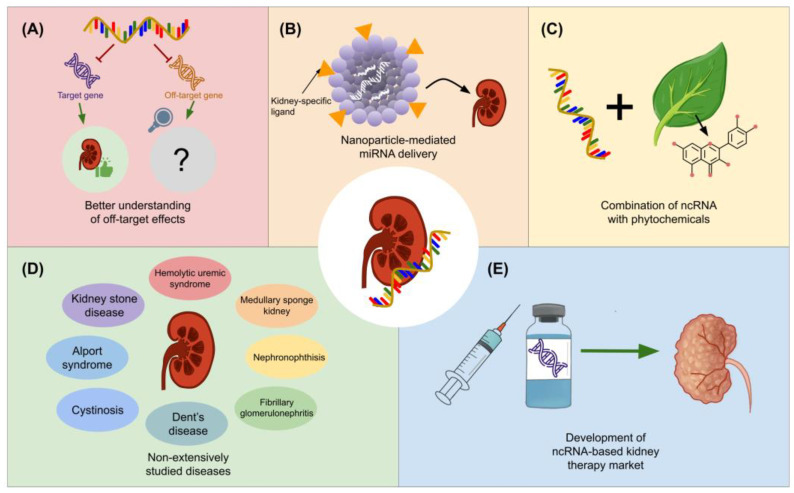
Future insights for miRNA- and lncRNA-based therapy development for kidney diseases. (**A**) It is anticipated that advances in the future toward understanding and managing off-target effects for ncRNA therapies will be beneficial for therapeutic applications. (**B**) NcRNA delivery, as another major challenge, could be addressed through the use of nanoparticles with kidney-specific ligands. (**C**) The combination of this therapeutic approach with phytochemical-based therapies could also lead to breakthroughs in the coming years. (**D**) Forthcoming research might also bring insights into renal diseases that have not extensively been explored from an ncRNA-based perspective. (**E**) All of these advancements, along with pre-clinical and clinical trials, might benefit the advancement of innovative RNA-based therapies against kidney diseases.

Additionally, when devising ncRNA-based drugs for kidney diseases, a pivotal consideration revolves around the potential of a single miRNA or lncRNA to concurrently influence multiple targets (e.g., mRNAs, miRNAs, chromatin regions, and other classes of RNAs), thereby bearing a broader impact on biological processes [144,145]. While the prospect of simultaneously impacting diverse cellular pathways holds promise for comprehensive disease management, it also presents challenges to maintaining specificity and minimizing unintended effects. Given the potential for pleiotropic actions of these ncRNAs, rigorous evaluation is highly required to guarantee the absence of unsought off-target effects in both disease-affected and healthy cells [146,147] (Figure 7A).

Achieving targeted delivery of ncRNA drugs to the kidneys represents a major hurdle in pharmaceutical development. One strategy to overcome such barriers could be nanoparticle-based carriers tailored to renal compartments. In such regard, nanoparticles can be engineered to possess sizes that optimize renal filtration, allowing them to pass through the glomerular filtration barrier and accumulate within the kidneys. To enhance the specificity of this approach, kidney-specific ligands (e.g., peptides and antibodies) can be attached to the nanoparticle’s surface. Consequently, those ligands will interact with the specialized receptors expressed on renal cells, facilitating the recognition and internalization of the ncRNA drug-loaded nanoparticles [33,148,149,150] (Figure 7B).

Along with the nanotechnology-based strategies, the concept of combining phytochemicals possessing therapeutic activity against kidney diseases with miRNA or lncRNA drugs holds promising potential for enhanced treatment outcomes, as previously demonstrated in managing myocardial infarction [151]. Co-delivering these compounds alongside miRNA or lncRNA using nanoparticles could be very effective. Some examples of phytochemicals that have been demonstrated to exhibit nephroprotective activity are quercetin, catechin, kaempferol, rutin, luteolin, and gallic acid [152] (Figure 7C). Meanwhile, forthcoming investigations should also explore the phytochemical-mediated regulation of ncRNA expression in kidney diseases since, to the best of our knowledge, such effects remain elusive. For instance, it has been reported that thymoquinone displays a nephroprotective effect against diclofenac-induced AKI through modulating the expression of miR-34a and mitofusin-2 (Mfn2) [153].

On the other hand, future research should analyze the prospective clinical applications of both miRNAs and lncRNAs to kidney diseases that have not yet been extensively studied in the arena of ncRNA drugs (Figure 7D). In this context, variations in the expression profile of miRNAs (e.g., miR-7b-3p, miR-22-3p, miR-127-3p, miR-181a-5p, miR-214-5p, and miR-223-3p) and lncRNAs (lnc-EVI5L-1, lnc-FAM72B-4, lnc-KIN-1, lnc-MB-6, lnc-SERPINI1–2, and lnc-TIGD1L2–3) have been found to be associated with kidney stones [154,155,156]. Moreover, some miRNAs and lncRNAs that have been reported to have prospective therapeutic relevance in kidney stone disease include miR-34a, miR-30c-5p, miR-204, LINC00339, and LINC01197 [157,158,159,160,161]. Similarly, recent outcomes support that anti-miR-21 can be applied to the treatment of Alport syndrome, a genetic disorder that impacts the glomerular basement membrane and leads to progressive kidney damage [162], while Lorenzen et al. [163] documented that the expression levels of miR-24 and miR-126 are altered in individuals with *Escherichia coli* O104:H4-induced hemolytic uremic syndrome and could be used as biomarkers. Even so, there are still several kidney diseases in which the regulatory functions of miRNAs and lncRNAs remain to be explored in depth, such as medullary sponge kidney, nephronophthisis, cystinosis, Dent’s disease, fibrillary glomerulonephritis, among others.

Undeniably, the approval of mRNA-based vaccines against COVID-19 has significantly bolstered the prospects of RNA-based therapeutics. This groundbreaking achievement has demonstrated the feasibility and safety of utilizing RNA molecules as powerful tools for therapeutic intervention [164]. As a result, this sphere of exploration is garnering heightened interest among investors, prompting the establishment of several pharmaceutical enterprises dedicated to the formulation of ncRNA-based therapies. Notable examples of such companies include Santaris Pharma, Regulus Therapeutics, miRagen Therapeutics, EnGeneIC, and Mirna Therapeutics Inc. [165]. In fact, as of now, two miRNA-based drugs for kidney diseases have already been entered into clinical trials, which are RG-012 (anti-miR-21 for Alport syndrome, NCT03373786) [166] and RGLS4326 (anti-miR-17 for polycystic kidney disease, NCT04536688) [167,168]. Hence, we speculate that the insights discussed in this present review will serve to fortify the investigation into the intricate interrelation between miRNAs, lncRNAs, and kidney diseases, thereby laying a foundation for the development of novel drugs poised for eventual clinical trials (Figure 7E).

## Data Availability

Not applicable.
